# Knowledge of cervical cancer and attendance at cervical cancer screening: a survey of Black women in London

**DOI:** 10.1186/1471-2458-14-1096

**Published:** 2014-10-22

**Authors:** Christine Ekechi, Adeola Olaitan, Rosie Ellis, Jacob Koris, Adaugo Amajuoyi, Laura AV Marlow

**Affiliations:** Department of Obstetrics & Gynaecology, Imperial College Healthcare Trust, London, W12 0HS England; University College London Hospital, 2nd floor North, 250 Euston Road, London, NW1 2PG England; University College London Medical School, 21 University Street, London, WC1E 6DE England; Leicester Royal Infirmary, Infirmary Square, Leicester, LE1 5WW England; Cancer Research UK Health Behaviour Research Centre, Department of Epidemiology & Public Health, University College London, Gower Street, London, WC1E 6BT England

**Keywords:** Cervical screening, Knowledge, Awareness, Race, Ethnicity

## Abstract

**Background:**

Women from ethnic minority backgrounds are less likely to attend cervical screening, but further understanding of ethnic inequalities in cervical screening uptake is yet to be established. This study aimed to explore the socio-demographic and ethnicity-related predictors of cervical cancer knowledge, cervical screening attendance and reasons for non-attendance among Black women in London.

**Methods:**

A questionnaire was completed by women attending Black and ethnic hair and beauty specialists in London between February and April 2013. A stratified sampling frame was used to identify Black hair specialists in London subdivisions with >10% Black population (including UK and foreign-born). Fifty-nine salons participated. Knowledge of cervical cancer risk factors and symptoms, self-reported screening attendance and reasons for non-attendance at cervical screening were assessed.

**Results:**

Questionnaires were completed by 937 Black women aged 18–78, describing themselves as being predominantly from African or Caribbean backgrounds (response rate 26.5%). Higher educational qualifications (p < .001) and being born in the UK (p = .011) were associated with greater risk factor knowledge. Older age was associated with greater symptom knowledge (p < .001). Being younger, single, African (compared to Caribbean) and attending religious services more frequently were associated with being overdue for screening. Women who had migrated to the UK more than 10 years ago were less likely to be overdue than those born in the UK. Of those overdue for screening who endorsed a barrier (67/133), ‘I meant to go but didn’t get round to it’ (28%), fear of the test procedure (18%) and low risk perception (18%) were the most common barriers.

**Conclusions:**

Ethnicity, migration and religiosity play a role in predicting cervical screening attendance among women from Black backgrounds. African women, those born in the UK and those who regularly attend church are most likely to put off attending. Additional research is needed to explore the attitudes, experiences and beliefs that explain why these groups might differ.

**Electronic supplementary material:**

The online version of this article (doi:10.1186/1471-2458-14-1096) contains supplementary material, which is available to authorized users.

## Background

Cervical cancer is the fourth most common cancer in women worldwide with a significant burden of mortality, particularly in developing countries [1]. In developed countries such as the US and UK incidence of cervical cancer is much lower, but disparities by socio-economic status, race and ethnicity remain [2–5]. These disparities are likely due to variation in risk factors for cervical cancer which include exposure to human papillomavirus, smoking status and non-attendance at cervical screening [6]. In England, an organised cervical screening programme dramatically reduces risk of cervical cancer, yet around 20% of women are not considered to be adequately screened [7].

A recent study that assessed a range of socio-economic variables, showed ethnicity was the most important socio-demographic predictor of having never attended cervical screening, with non-white women more than twice as likely to have never attended [8]. The term ethnicity refers to “the group to which people belong… as a result of certain shared characteristics including geographical and ancestral origins, but particularly cultural traditions and languages” [9]. A multi-faceted concept, ethnicity encompasses a range of factors such as migration history, language and religion. To date the influence that these factors have on cervical screening uptake has not been explored. Qualitative studies suggest lower knowledge of cervical cancer among minority groups could explain low engagement with cancer prevention services, [10] but this has not been explored quantitatively.

Identifying ethnic groups with poor knowledge of cervical cancer and low engagement with cancer prevention services would highlight where interventions to reduce inequalities need to be directed. A number of studies have examined factors associated with screening uptake in British South Asian women, [11] but to our knowledge there are no studies exploring cervical cancer knowledge or screening behaviour among Black women in the UK. In 2011, the Black population in England was approximately 1.8 million, including predominantly those from African and Caribbean backgrounds (UK-born and foreign-born) [12]. Women from African and Caribbean backgrounds are often grouped together for research purposes, but there is evidence of variation between these ethnic subgroups in other behavioural risk factors for cervical cancer (smoking status and sexual behaviour [13, 14]). Differences in cervical screening uptake have not been explored.

We conducted a community-based survey of Black women in London. Our objectives were i) to explore socio-demographic and ethnicity-related factors associated with cervical cancer knowledge and non-attendance at cervical screening and ii) consider self-reported reasons for non-attendance at screening.

## Methods

A questionnaire, examining knowledge and attitudes to cervical cancer screening was distributed to Black and ethnic hair and beauty specialists in London. We decided to focus data collection in London because census data (2011) show that more than half of England’s Black population reside in London [12]. We planned to include all women over 18 years old, UK-born and foreign-born who classified themselves as African, Caribbean, any other Black/African/Caribbean background, mixed White and Black Carribbean, mixed White and Black African (in response to the 2011 census question on ethnic group). We felt that recruiting through hair and beauty salons serving the Black community would help us identify women from our target population including those who may not routinely access healthcare. Similar methods have been used for community-based recruitment of ethnic minority groups in the United States [15].

### Recruitment

The aim was to distribute 6000 questionnaires through 100 establishments over a twelve week period. Initially, a stratified sampling frame was developed as follows, 1) London boroughs^a^ with a greater than 10% proportion of residents from Black backgrounds (including UK and foreign born) were selected, 2) The number of salons to be recruited within each borough was determined according to the size of the Black population in that borough, 3) A list of hair and beauty salons catering to the Black community within each of the selected boroughs were identified using an internet search (122 salons were identified). We intended to target 100 Salons across 18 boroughs, between 3 and 10 salons per borough (see Additional file 1). Where listed establishments had moved location or were unwilling to participate in the study, alternative local salons within the same borough were approached. Many of the salons on the original internet-based recruitment list were unavailable so a secondary recruitment method was based on identifying local high streets (focal points for business, particularly shops) in each borough and visiting these to look for salons. In some instances hair salons who agreed to participate would suggest other salons that might be interested in taking part. For logistical reasons, recruitment ceased after the allocated recruitment period (12 weeks) despite not reaching our original target of 100 salons.

Questionnaires were distributed between February and April 2013. Each participating salon was visited (by RE and/or AA) and left with 60 self-administered questionnaires to distribute to all women who attended over a one week period (7 days inclusive). Salons were given key rings to offer their clients as a thank you for participating. Some hair salons were visited on more than one occasion if at first visit it was apparent that the initial response rate was low. Questionnaires were completed anonymously, were sealed in an envelope provided and deposited in a secure box.

All women over the age of 18 who described themselves as being from a Black background were eligible for the study. Although women are not invited to cervical screening in England until they are 25 years old, we felt it was appropriate to assess knowledge of cervical cancer among women in a wider age range.

### Measures

A self-administered structured questionnaire was developed based on previous research on cervical cancer knowledge and screening (Additional file 2). Socio-demographic and ethnicity-related variables were assessed using items from the 2001 Census. We assessed ethnicity, country of birth, length of time in the UK, language spoken at home, religion and religiosity. We also assessed education level, marital status and smoking status. Education was recoded into low (no formal qualifications or General Certificate of Secondary Education), medium (vocational qualifications or A-levels) or high (a university degree).

Knowledge questions were taken from the Cervical Cancer Awareness Measure (Cervical CAM) a validated tool to measure awareness of cervical cancer [16]. Two open questions from the Cervical CAM were used to examine knowledge of cervical cancer risk factors and symptoms. Participants freely listed risk factors and symptoms, and their responses were coded in accordance with a predefined list of target risk-factors and symptoms identified in the scientific literature and forming the National Health Service (NHS) Department of Health’s ‘Key Messages’ on cervical cancer. Only correct risk factors and symptoms were coded.

To assess attendance at cervical screening women were asked “Which of these statements, if any, describes whether you have had cervical screening (smears)?” with the following response options: I have had a test within the last 3 years; My last test was 3 to 5 years ago; My last test was more than 5 years ago; I have never been invited to have a test; I have been invited but have never had a test; I have had a hysterectomy so I don’t need to have tests; I have never heard of cervical screening; I am too young to be invited (18–24 years); None of the above. Women who had never had a smear test, or did not attend their last screening were asked to indicate why by selecting from a predefined list. The cervical screening attendance question and the predefined list of barriers had been used previously in a population-representative survey [17].

### Analysis

Data were analysed using SPSS v.20. Overall risk factor knowledge and symptom knowledge scores were calculated by allocating a score of ‘1’ for each cited item that corresponded with the pre-defined list. Participants who did not cite any items on this list were allocated a score of ‘0’. The same method was used in a previous study [18]. We have reported the means for these scores, but because the data were highly skewed participants were recoded into two groups, those who ‘cited’ vs ‘did not cite’ at least 1 correct risk factor/symptom. We used logistic regression to explore associations between each socio-demographic/ethnicity-related variable and citing a correct risk factor/symptom. Logistic regression was also used to explore predictors of being overdue for screening. Women were considered ‘overdue’ for screening if they were 25–49 years and had not been screened in the last 3 years or if they were 49–64 and had not been screened in the last 5 years. Odds ratios (OR) and 95% confidence intervals (CI) are reported.

## Results

A total of 3540 questionnaires were distributed through 59 Salons and 937 were returned (response rate - 26.5%). Blank questionnaires were excluded (n = 40), as were those with no completed demographic information provided (n = 21). Missing demographic data for the remaining 876 women was approximately 5% for most variables, but slightly higher for birthplace and migration year (8% and 10%). Participants were only excluded from analyses involving variables where their data was missing.

### Sample characteristics

Sample characteristics are shown in Table 1. Over half the women described themselves as Caribbean (56%) and a quarter as African (25%), with the rest describing their background as mixed White and Black Caribbean (5%), mixed White and Black African (3%) or other Black (4%). Most spoke English as their main language (97%). Most were born in the UK (61%), but many of these women had a least one parent born outside the UK (84%). A third of women were born outside of the UK, but most of these women had been living in the UK for more than 10 years. The majority were Christian (77%), with 41% attending religious service at least once a month. Almost half were single (48%) and 50% had a high level of educational qualifications.

**Table 1:** **Sample characteristics**

	Whole sample (n = 876)		Screen eligible (n = 652)	
	N	%*	N	%*
**Marital status**				
Single	417	47.6	295	45.1
Married	214	24.4	184	28.4
Cohabiting	95	10.8	77	11.8
Divorced/separated/widowed	106	12.1	94	14.4
**Education**				
Low level	131	15.0	100	15.3
Medium level	240	27.4	162	24.8
High level	436	49.8	348	53.4
Other	30	3.4	25	3.8
**Ethnicity**				
Caribbean	488	55.7	380	58.3
African	216	24.7	166	25.5
Mixed white and Black Caribbean	39	4.5	33	5.1
Mixed white and Black African	25	2.9	20	3.1
Any other Black background	33	3.8	28	4.3
Other mixed background	13	1.5	9	1.4
Other	18	2.1	11	1.7
**Birthplace**				
UK-born and parents UK-born	83	9.5	46	7.1
UK-born, 1 parent UK-born	204	23.3	159	24.4
UK born, no parents UK-born	244	27.9	205	31.4
Not UK-born	275	31.4	221	33.9
**Year moved to UK**				
1956-1972	49	5.5	35	5.4
1973-1990	69	7.9	62	9.5
1991-2002	95	10.8	77	11.8
2003-2013	44	5.0	34	5.2
**Religion**				
Christian	678	77.4	498	76.4
Muslim	27	3.1	23	3.5
No religion	103	11.8	84	12.9
Other	27	3.1	25	3.8
**Religious service attendance**				
Rarely or never	238	27.2	181	27.8
Few times a year	239	27.3	180	27.6
1-3 times a month	103	11.8	75	11.5
At least once a week	251	28.7	192	29.4
**Smoking Status**				
I have never smoked	508	58.0	379	58.1
I used to smoke	210	24.0	162	24.8
I currently smoke	137	15.6	101	15.5

*Unaccounted percentage is missing data.

### Knowledge of cervical cancer symptoms and risk factors

Just over half of women cited at least one cervical cancer risk factor (55%, n = 481) and 58% (n = 509) cited at least one cervical cancer symptom. While 41% of women cited both a risk factor and a symptom, 28% could not cite either. The percentage of women citing each risk factor/symptom is shown in Table 2.

**Table 2:** **Cervical cancer risk factor and symptoms knowledge (n = 876)**

	N	%*
**Risk factors for cervical cancer**		
Having many sexual partners	132	15.1
Smoking	102	11.6
Not going for regular smear tests	89	10.2
Unprotected sex	57	6.5
Infection, STI/STD or virus	48	5.5
Starting to have sex at a young age	44	5.0
Infection with Human papillomavirus	38	4.3
Long term use of the contraceptive pill	17	1.9
Having many children	5	0.6
Having a weakened immune system	3	0.3
**Symptoms of cervical cancer**		
Vaginal bleeding between periods	282	32.2
Abnormal discharge	219	25.0
Persistent pelvic/abdominal pain	145	16.6
Pain/discomfort during sex	78	8.9
Vaginal bleeding during/after sex	56	6.4
Persistant lower back pain	13	1.5
Blood in stool/urine	13	1.5
Unexplained weight loss	12	1.4
Heavier/longer periods than normal	6	0.7
Vaginal bleeding after the menopause	4	0.5

*Percentage of entire sample; Women were able to select more than one risk factor/symptom.

Note: Risk factors/Symptoms identified in the NHS Department of Health’s ‘Key Messages’ on cervical cancer [18].

The most commonly cited risk factors were, having many sexual partners (15%), smoking (12%) and not going for smear tests (10%). Only 4% cited infection with human papillomavirus, but 29% cited at least one risk factor relating to sexual behaviour (having multiple sexual partners, sex at a young age, unprotected sex, HPV or other STIs). On average women cited 0.61 correct risk factors (range 0–6). Citing at least one correct risk factor was not associated with ethnicity, length of time in the UK, religion or religiosity, however women who were born in the UK were more likely to cite a risk factor than women born outside the UK (OR = 1.46, CI: 1.09-1.96). Women with a high level of education were more likely to cite a risk factor than those with a low level of education (OR = 2.22, CI: 1.46-3.35). Age and marital status were not significantly associated with risk factor knowledge. In adjusted analyses, including education and being born in the UK, both variables remained significant.

The most commonly cited symptoms were vaginal bleeding in-between periods and unusual vaginal discharge (cited by 32% and 25% of women respectively). Less than 1% of women cited heavier/longer periods or vaginal bleeding after the menopause. On average women cited 0.95 symptoms (range 0-5). Age was the only variable significantly associated with citing at least one correct symptom (p < .001). Compared to 16–24 year olds each older age group was progressively more likely to cite a correct symptom: 25–34 years (OR = 2.54, CI: 1.55-4.18), 35–44 years (OR = 2.76, CI: 1.66-4.57), 45–64 years (OR = 4.52, CI: 2.73-7.49). Women over 65 years were no more likely to cite a correct symptom than the youngest group (p > .05). Symptom knowledge was not associated with ethnicity, being born in the UK, religion or religiosity or education. Marital status was associated with citing a correct symptom, but this was no longer the case when adjusting for age.

### Attendance at screening

Of the eligible population (those aged 25–64 years, who had not had a hysterectomy, n = 652), 75% had been screened in the last 3 years and 16% in the last 3–5 years. Sample characteristics for the screen eligible women are shown in Table 1. Most women were last screened at their GP surgery (75%), but health centres (10%), hospitals (7%) and private clinics (2%) were used by some women. Overall, 20.4% of women were overdue for screening (n = 133). In univariable analyses (see Table 3) women were more likely to be overdue screening if they were younger (23% of 25–34 year olds and 25% of 35–44 year olds compared to 13% of 45–64 year olds, p = .007 and .003 respectively) and single (27% compared with 15% of married/cohabiting women, p = .001). Women who considered themselves to be African were more likely to be overdue (26% compared with 18% of those who considered themselves to be Caribbean, p = .024). Women who had migrated to the UK more than 10 years ago were less likely to be overdue for screening than women born in the UK (12% compared with 23%, p = .003). Women who attended religious services at least once a week were more likely to be overdue than those who rarely or never attended (27% compared with 17%, p = .020). Education level and religion were not significantly associated with screening status. Women who cited at least one symptom were less likely to be overdue for screening (OR = 0.58, CI: 0.40-0.85). In a multivariable model all variables remained significant except for ethnicity and symptom knowledge (see Table 3).

**Table 3:** **Associations between socio-demographic variables and being overdue for screening**

		Univariable analyses	Multivariable analyses^1^
	Overdue n (%)	OR [95% CI]	OR [95% CI]
**Age Group**			
25-34	54 (23.3)	1.98 [1.21-3.25]**	1.35 [0.75-2.42]
35-44	50 (24.9)	2.16 [1.32-3.58]**	1.92 [1.09-3.37]*
45-64	29 (13.3)	1.00	
**Marital Status**			
Single	79 (26.9)	2.03 [1.33-3.10]**	2.11 [1.32-3.37]**
Married/Cohabiting	40 (15.3)	1.00	
Divorced/separated/widowed	81 (13.8)	0.89 [0.45-1.74]	0.98 [0.45-2.14]
**Education**			
Low level	15 (15.0)	0.67	
Medium level	37 (22.8)	1.13 [0.37-1.24]	
High level	72 (20.7)	1.00 [0.72-1.77]	
**Ethnicity**			
Caribbean	74 (17.9)	1.00	
African	48 (25.9)	1.61 [1.06-2.44]*	1.59 [0.97-2.60]
Other	9 (19.6)	1.12 [0.52-2.42]	1.29 [0.57-2.92]
**Birthplace**			
Born in the UK	95 (23.2)	1.00	
Migrated <10 years ago	11 (32.4)	1.59 [0.75-3.37]	1.01 [0.44-2.29]
Migrated >10 years ago	21 (12.1)	0.46 [0.28-0.76]**	0.42 [0.24-0.75]**
**Religion**			
Christian	109 (21.9)	1.00	
Muslim	6 (26.1)	1.26 [0.48-3.26]	
No religion	11 (13.1)	0.54 [.275-1.05]	
**Religious service attendance**			
Rarely or never	60 (16.6)	1.00	
Few times a year	36 (20.0)	1.26 [0.74-2.15]	1.27 [0.72-2.26]
1-3 times a month	11 (14.9)	0.88 [0.42-1.86]	1.04 [0.48-2.28]
At least once a week	51 (26.6)	1.82 [1.10-3.02]*	2.05 [1.16-3.54]*
**Risk factor knowledge score**			
0	78 (22.5)	1.00	
1+	55 (18.0)	0.76 [0.51-1.11]	
**Symptom knowledge score**			
0	68 (25.8)	1.00	
1+	65 (16.8)	0.58 [0.40-0.85]**	0.72 [0.47-1.09]

^1^Includes: age group, marital status, ethnicity, birthplace, religious service attendance, Symptom knowledge score.

*p < .05; **p < .01.

### Reasons for non-attendance in those overdue for screening

Of the 133 women who were overdue for screening, half selected at least one reason for not attending from the list provided (n = 67, see Table 4). Younger women (χ^2^(2) = 6.54, p = .038), single women (χ^2^(2) = 8.05, p = .018) and those with the highest level of education (χ^2^(2) = 12.06, p = .002) were more likely to provide reasons for non-attendance, but there were no differences by ethnicity, birthplace, or religiosity. Of those who selected a reason for never being screened or delaying screening, ‘I meant to go but didn’t get round to it’ (28%), fear of the test procedure (18%) and low risk perception (18%) were the most common responses. Table 4 shows the reasons women endorsed by ethnicity, birthplace and religiosity, but numbers were too small to analyse this statistically.

**Table 4:** **Reasons for non-attendance among women overdue for screening (number endorsed, per cent in brackets)**

		Ethnicity		Birthplace		Religiosity*		
	Overall (n = 67)	Caribbean (n = 35)	African (n = 29)	UK born (n = 20)	Not UK born (n = 47)	None/low (n = 18)	Medium (n = 21)	High (n = 27)
**Practical barriers**								
I meant to go but didn’t get around to it	19 (28.4)	11 (31.4)	8 (27.6)	11 (23.4)	8 (40.0)	6 (33.3)	5 (23.8)	7 (25.9)
It is difficult to get an appointment at a time that suits me	11 (16.4)	9 (25.7)	1 (3.4)	11 (23.4)	0 (0)	3 (16.7)	4 (19.0)	4 (14.8)
Too busy/inconvenient	9 (13.4)	5 (14.3)	4 (13.8)	5 (10.6)	4 (20.0)	4 (22.2)	1 (4.8)	4 (14.8)
**Emotional Barriers**								
Fear of the test procedure	12 (17.9)	3 (8.6)	9 (31.0)	9 (19.1)	3 (15.0)	1 (5.6)	5 (23.8)	6 (22.2)
Fear of a ‘bad’ result	10 (14.9)	5 (14.3)	5 (17.2)	6 (12.8)	4 (20.0)	3 (16.7)	4 (19.0)	3 (11.1)
Bad experience of cervical screening in the past	6 (9.0)	3 (8.6)	3 (10.3)	6 (12.8)	0 (0)	4 (22.2)	1 (4.8)	1 (3.7)
Embarrassment	6 (9.0)	2 (5.7)	4 (13.8)	5 (10.6)	1 (5.0)	1 (5.6)	2 (9.5)	3 (11.1)
**Beliefs about screening**								
I do not believe I am at risk	12 (17.9)	4 (11.4)	7 (24.1)	9 (19.1)	3 (15.0)	5 (27.8)	1 (4.8)	6 (22.2)
I do not believe the test is needed	4 (6.0)	0 (0)	3 (10.3)	2 (4.3)	2 (10.0)	2 (11.1)	1 (4.8)	1 (3.7)
**Other**								
I have never been invited for screening	9 (13.4)	4 (11.4)	5 (17.2)	5 (10.6)	4 (20.0)	1 (5.6)	3 (14.3)	5 (18.5)
I didn’t understand the screening invitation letter	1 (1.5)	1 (2.9)	0	1 (2.1)	0 (0)	1 (5.6)	0 (0)	0 (0)

*None/low: Rarely or never; Medium: 1-3 times a month/A few times a year; High: At least once a month.

## Discussion

We explored socio-demographic predictors of cervical cancer knowledge and cervical screening behaviour among Black women living in London.

For cervical cancer knowledge, education level was the most important predictor of citing a risk factor, while age was the most important predictor of citing a symptom, consistent with other studies in the cervical cancer context [18, 19]. To the most part, none of the ethnicity-related variables were associated with knowledge, with the exception being migration status. In general, the most commonly reported risk factors (many sexual partners and smoking) and symptoms (unusual vaginal bleeding and persistent unusual discharge) in our study and the population-representative study were the same [18]. Interestingly, persistent pain was more commonly cited as a symptom in our study (17% compared with 1%) and this could be a cause for concern if it means symptoms in the absence of pain are not taken seriously by women from Black backgrounds. Previous work in the context of breast cancer suggests some African American women feel confused over whether pain is a symptom of cancer [20] and this warrants further investigation.

We also explored non-attendance at cervical screening. Younger age and being single were associated with being overdue for screening, consistent with other studies [17, 21, 22]. Many of the ethnicity-related variables were also associated with being overdue for screening. The percentage of those who were overdue for cervical screening was higher in those who had recently migrated to the UK (32%), but much lower in those who had been in the UK for more than 10 years (12%). This is consistent with studies in the US that have shown immigrants are less likely to have been screened but this inequality decreases with acculturation (length of time in the new country) [23, 24]. There were also differences between subgroups of the Black population, with women from African backgrounds more likely to be overdue for screening than Caribbean women. This is interesting when considered in the context of other cervical cancer risk factors (smoking and having multiple sexual partners) which suggest that African women may be at lower risk of cervical cancer than Caribbean women [13, 14]. Agyemang et al. [25] argued that health researchers should consider diversity within Black populations because differences in beliefs and behaviours exist, yet there remains a paucity of research exploring differences in cancer beliefs between African and Caribbean women in the UK.

Non-attenders reported not getting round to screening, difficulty making an appointment, fear of the test procedure and fear of a bad result as barriers to screening. African women who were overdue screening were more likely to endorse fear of the test and embarrassment and to believe they were not at risk of cervical cancer. Small numbers mean we could not run statistical analyses on this data so this needs further exploration. Similar to other studies with Black women we found an association between symptom knowledge and screening behaviour [26] which could suggest interventions designed to increase awareness might consequently have a positive influence on behaviour. Alternatively, cervical screening could be acting as an opportunity for women to learn about cervical cancer risk factors and symptoms.

### Strengths and limitations

To our knowledge this is the first study to explore cervical cancer knowledge and screening behaviour with a focus on Black women living in London. We took a novel approach to recruitment, targeting a specific ethnic group by approaching women in the community. We felt that targeting women through hairdressers, a non-health care setting would help us recruit those who do not always engage with health care. Using this method, we successfully recruited a large number of women with a good range on the ethnicity-related variables. We used questions from validated measures used in previous population-based surveys, although we did not pilot our survey before implementing the study.

This study has some limitations. The response rate was low (26.5%), so we cannot assume our findings are generalisable to the entire population of women from Black ethnic backgrounds living in London. In comparison to 2011 census statistics for the London boroughs we sampled from, we recruited more Black Caribbean women (58% vs 29%) and fewer African women (25% vs 45%), with fewer women born outside the UK (31% vs 46% - figure for women of all ages). One reason for this could be that the survey was completed in written English, thereby excluding women who cannot read English, who are more likely to born outside of the UK and be from African backgrounds. Around half of our sample had degree-level qualifications and this is also higher than population-level figures: 40% for men and women from Black African and 26% from Black Caribbean backgrounds. Five-yearly screening uptake in our study was 91%, which is higher than the national figure (80%) and we acknowledge that this suggests non-attenders were under-represented. However, more women were overdue for screening in this study than in a recent population-representative survey which used ‘gold-standard’ recruitment methods [17]. It is also possible that by relying on self-reported screening attendance our findings are subject to social desirability bias, despite our attempts to minimise this by ensuring anonymity. These limitations have important implications and suggest that the overall percentages we report should be interpreted with caution.

Our decision to focus recruitment in London means we cannot necessarily extrapolate our findings to Black communities in other parts of the UK, both rural and urban. Recruiting through hair salons means women who do not attend salons frequently or at all, perhaps the more socially isolated or those from lower socio-economic backgrounds, are less likely to be included. We also relied on salon staff to distribute and collect the questionnaire (the denominator for the response rate is the number of questionnaires distributed to salons rather than the number of women approached). Difficulties with identifying and engaging salons meant we had to adapt our methodology and ultimately did not reach our initial recruitment target. We feel our experience may have important implications for other researchers considering a similar community-based approach to survey recruitment and have provided more detail about our experience (see Additional file 3).

## Conclusions

This study suggests that ethnicity, migration and religiosity play a role in predicting cervical screening attendance among Black women in London. Although previous work had suggested ethnic inequalities in cervical screening uptake between white and non-white women, [8] this is the first study to suggest that among Black women those from African backgrounds, and those who attend religious service on a frequent basis could be the most likely to delay attending cervical screening. More research is needed to explore the attitudes, experiences and beliefs that might explain why these groups differ.

### Details of ethical approval

The study was approved by the UCL research ethics committee (ref:0496/011).

### Endnote

^a^Boroughs are subdivisions of greater London, each governed by a different council. London has 32 boroughs, each with a population of approximately 150,000-363,000 [27].

## Electronic supplementary material

Additional file 1: **Sampling frame.** (DOCX 17 KB)

Additional file 2: **Questionnaire: Attitudes to cervical screening among women attending specialist black hairdressers in London.** (DOCX 48 KB)

Additional file 3: **Difficulties with recruitment.** (DOCX 15 KB)
