# Supplementary material for: Knowledge of cervical cancer and attendance at cervical cancer screening: a survey of Black women in London
Source: BMC Public Health. 2014 Oct 22;14:1096. doi: 10.1186/1471-2458-14-1096 (PMC4216339; doi:10.1186/1471-2458-14-1096)
Supplement: Supplementary file 1 — Additional file 1: Sampling frame. (DOCX 17 KB) [file 12889_2014_7195_MOESM1_ESM.docx]

**Supplementary material 1: Sampling Frame**

|  | Total  Population (n) | Total  Black population (n) | % population from black backgrounds | Recruitment target  (# of salons) | Salons recruited  (# of salons) | Questionnaires returned |
| --- | --- | --- | --- | --- | --- | --- |
| **High % of population from black backgrounds:** |  |  |  |  |  |  |
| Lewisham | 282400 | 90400 | 32% | 9 | 6 | 168 |
| Lambeth | 308600 | 82700 | 27% | 8 | 6 | 131 |
| Newham | 292300 | 75500 | 26% | 7 | 2 | 9 |
| Southwark | 306200 | 79000 | 26% | 7 | 6 | 73 |
| Hackney | 242600 | 61900 | 26% | 7 | 3 | 34 |
|  |  |  |  |  |  |  |
| **Medium % of population from black backgrounds:** |  |  |  |  |  |  |
| Croydon | 352300 | 83900 | 24% | 7 | 2 | 9 |
| Brent | 287800 | 62800 | 22% | 6 | 6 | 51 |
| Barking and Dagenham | 193400 | 42200 | 22% | 6 | 1 | 6 |
| Greenwich | 264400 | 59000 | 22% | 6 | 5 | 17 |
| Waltham Forest | 234500 | 48700 | 21% | 6 | 3 | 113 |
| Haringey | 247500 | 51700 | 21% | 6 | 5 | 61 |
|  |  |  |  |  |  |  |
| **Low % of population from black backgrounds:** |  |  |  |  |  |  |
| Enfield | 297300 | 52200 | 18% | 5 | 3 | 38 |
| Hammersmith and Fulham | 181200 | 23400 | 13% | 4 | 2 | 23 |
| Redbridge | 265500 | 32800 | 12% | 4 | 2 | 19 |
| Islington | 211500 | 25500 | 12% | 3 | 2 | 58 |
| Merton | 198700 | 22200 | 11% | 3 | 0 | 0 |
| Ealing | 325200 | 31800 | 10% | 3 | 1 | 2 |
| Camden | 218800 | 21200 | 10% | 3 | 4 | 63 |
|  |  |  |  |  |  |  |

NB - High % = 25%+, Medium % = 20-24%, Low percentage = 10-19%

Figures based on ethnic group population projections provided by the Greater London Authority, 2010.
