# Supplementary material for: Knowledge of cervical cancer and attendance at cervical cancer screening: a survey of Black women in London
Source: BMC Public Health. 2014 Oct 22;14:1096. doi: 10.1186/1471-2458-14-1096 (PMC4216339; doi:10.1186/1471-2458-14-1096)
Supplement: Supplementary file 2 — Additional file 2: Questionnaire: Attitudes to cervical screening among women attending specialist black hairdressers in London. (DOCX 48 KB) [file 12889_2014_7195_MOESM2_ESM.docx]

**Attitudes to Cervical Screening among Women attending Specialist Black Hairdressers in London**

Dear Salon User,

We would be most grateful if you could take a few minutes of your time to complete this questionnaire. We are interested in attitudes to cervical screening (smear tests) among Black and ethnic minority women. Some research has shown that women from Black and ethnic minority backgrounds are less likely to go for cervical screening, we are trying to understand why this might be.

**Who is carrying out this survey?**

This survey is being carried out by researchers at University College London and UCLH. The information we will get from this survey will help us to design better ways of getting information to you and your community.

**Why am I being asked to participate in this survey?**

We are asking all women over 18 years of age, attending specialist Black Hairdressers in London this week to help us by completing this questionnaire. If you are under 18, please do not complete this questionnaire. Simply hand it back to the salon staff.

**About the questionnaire**

The questionnaire asks general questions about you and questions about your feelings about cervical screening. Your answers are completely anonymous which means that the researchers will not be able to identify you but all the information you give will be extremely valuable. It will take no more than 10 minutes to answer the questions. When you have completed the questionnaire, please put it in the envelope provided, seal it and put it in the sealed collection box. A report of the study findings will be available on request.

**What if I do not wish to participate?**

You do not have to take part and even if you start completing the questionnaire you can change your mind at any time. Submission of a completed questionnaire implies consent to participate. If you do not wish to fill in the questionnaire please let us know why in the space below and put this in the envelope provided.

**I do not wish to fill in this questionnaire because:**

Thank you for your help.

Adeola Olaitan

Consultant Gynaecologist, UCLH, London

Laura Marlow

Post-doc Research Fellow, UCL

This study has been approved by the UCL Research Ethics Committee (Project ID Number): 0496/011 For enquiries or to request a final report please contact: l.marlow@ucl.ac.uk; 0207 679 1798

| **A: Your experience of cervical cancer screening (smear tests)** | |
| --- | --- |
| **To start off with, we would like to know a bit about your experience of going for cervical screening (smear tests)** | |
| **A1: Women in England are invited to have cervical screening (smear tests) every 3-5 years from age 25 to age 64. Which of these statements, if any, describes whether you have had cervical screening (smears)?** | |
| □ I have had a test within the last 3 years  □ My last test was 3 to 5 years ago  □ My last test was more than 5 years ago  □ I have never been invited to have a test  □ I have been invited but have never had a test | □ I have had a hysterectomy so I don't need to have tests  □ I have never heard of cervical screening  □ I am too young to be invited (18-24 years)  □ None of the above |
| **A2: Did you attend your last invitation for cervical screening?** | |
| □ Yes  □ No | □ Don’t know  □ I’ve never had an invitation |
| **A3: Where did you attend your last cervical screening?** | |
| □ My GP surgery  □ A Hospital  □ A Health Centre | □ A Private Clinic  □ Other (please state) ………………………….  □ N/A – I’ve never had one |
| **A4: If you have never had a smear teat or did not attend your last screening, please indicate why (You may choose more than one response)** | |
| □ Fear of the test procedure  □ Fear of a ‘bad’ result  □ Bad experience of cervical screening in the past  □ Embarrassment  □ I do not believe that I am at risk  □ I do not believe the test is needed  □ It was difficult to make an appointment at a time that suited me  □ I didn’t understand the screening invitation letter | □ The test is culturally/ religiously unacceptable  □ Too busy/ inconvenient  □ I meant to go but didn’t get round to it  □ I have had a hysterectomy  □ I have been treated for a gynaecological cancer  □ I have never been invited for screening  □ I am under 25 years  □ Other (please state) …………………………. |

|  | | | | | |
| --- | --- | --- | --- | --- | --- |
| **A5: Have you ever had an abnormal smear result (sometimes called pre-cancerous changes)?** | | | | | |
| □ Yes  □ No [GO TO A9] | | | □ Don’t know [GO TO A9]  □ I’ve never had an invitation [GO TO A9] | | |
| If your answer to this question was *no*, *don’t know* or *I’ve never had an invitation* then  PLEASE GO TO QUESTION A9 | | | | | |
| **A6: Would you say that your experience of having an abnormal smear result has put you off cervical screening in the future?** | | | | | |
| □ Definitely not | □ Probably not | □ Not sure | | □ Yes, probably | □ Yes, definitely |
| **A7: Have you ever had further investigation following an abnormal cervical screening result (a colposcopy)?** | | | | | |
| □ Yes  □ No [GO TO A9] | | | □ Don’t know [GO TO A9] | | |
| If your answer to this question was *no* or *don’t know* then  PLEASE GO TO QUESTION A9 | | | | | |
| **A8: Would you say that your experience of having further investigation following an abnormal smear result has put you off cervical screening in the future?** | | | | | |
| □ Definitely not | □ Probably not | □ Not sure | | □ Yes, probably | □ Yes, definitely |
| **A9: Would you recommend cervical screening to a female friend/ relative?** | | | | | |
| □ Definitely not | □ Probably not | □ Not sure | | □ Yes, probably | □ Yes, definitely |
| Please tell us why you have given this answer  ………………………………………………………………………………………………………………………………………………………. | | | | | |

| **B: Your knowledge and attitudes to cervical cancer screening** | | | | | |
| --- | --- | --- | --- | --- | --- |
| **In this section we would like to know a bit about your knowledge of and your attitudes towards cervical cancer screening (smear tests)** | | | | | |
| **B1: Do you understand the reason(s) for cervical screening?** | | | | | |
| □ Yes  □ No | | □ Don’t know | | | |
| **B2: There are many warning signs and symptoms of cervical cancer. Please name as many as you can think of:** | | | | | |
| …………………………………………………………………………………………………………………………………………………………..  ………………………………………………………………………………………………………………………………………………………….. | | | | | |
| **B3: What things do you think affect a woman’s chance of getting cervical cancer?** | | | | | |
| …………………………………………………………………………………………………………………………………………………………..  ………………………………………………………………………………………………………………………………………………………….. | | | | | |
| **B4: Thinking of information concerning “female health”, which of these are the main sources of information you normally use to find out about such issues?** | | | | | |
| □ My Friends or family  □ My GP  □ Health magazines  □ Other magazines | □ Television  □ The Internet  □ Health Charities  □ Other (please state)  ……………………………… | | | | |
| **B5: Would you say the media coverage of cervical cancer and the unfortunate death of Jade Goody;** | | | | | |
|  | | | **Yes** | **No** | **Don’t know** |
| … increased your awareness of the disease? | | | □ | □ | □ |
| … encouraged you to seek a cervical screening test? | | | □ | □ | □ |
| … had no affect on you? | | | □ | □ | □ |

| **B6: We would like to know about your attitudes to cancer screening tests. Please indicate how much you agree with the following items:** | | | | | |
| --- | --- | --- | --- | --- | --- |
|  | Strongly disagree | Disagree | Not sure | Agree | Strongly agree |
| Cervical smear tests are not good at detecting cervical cancer in its early stages | □ | □ | □ | □ | □ |
| Having a regular smear test is not a good idea | □ | □ | □ | □ | □ |
| If I have regular smear tests, cervical cancer will be found before it is advanced | □ | □ | □ | □ | □ |
| Having a smear test would not give me peace of mind | □ | □ | □ | □ | □ |
| I have a lot to gain by having regular smear tests | □ | □ | □ | □ | □ |
|  |  |  |  |  |  |
| I think about the future and this influences my behaviour today | □ | □ | □ | □ | □ |
| I'm prepared to make sacrifices now for benefit in the long run | □ | □ | □ | □ | □ |
| I leave the future to take care of itself | □ | □ | □ | □ | □ |
| I prefer to think of the 'here and now' rather than the future | □ | □ | □ | □ | □ |
| It is possible that I will get cervical cancer in the future | □ | □ | □ | □ | □ |
| I believe that cervical cancer is serious | □ | □ | □ | □ | □ |
|  |  |  |  |  |  |
| I may one day be at risk of getting cervical cancer | □ | □ | □ | □ | □ |
| I believe that cervical cancer has serious negative consequences | □ | □ | □ | □ | □ |
| It is likely that I will get cervical cancer one day | □ | □ | □ | □ | □ |
| I believe that cervical cancer can be extremely harmful | □ | □ | □ | □ | □ |
| Serious diseases like cancer are all fated; we cannot prevent them from happening | □ | □ | □ | □ | □ |
| If you are fated to get cancer, you will get cancer; there is nothing you can do to change fate | □ | □ | □ | □ | □ |
| If you don't die from this, you'll die from that, so there's no point in taking screening tests | □ | □ | □ | □ | □ |

| **C: About you and your health** | | | | | | |
| --- | --- | --- | --- | --- | --- | --- |
| **Finally, we would like to ask you a few questions about you and your health, this will help us to analyse the results of the questionnaire.** | | | | | | |
| **C1: What is your age?** | | | | | | |
| ………….. | | | |  | | |
| **C2: What is your marital status?** | | | | | | |
| Single (never married) | Married | Cohabiting (living with partner) | | | Divorced or separated | Widowed |
| □ | □ | □ | | | □ | □ |
| **C3: Which ethnic group do you consider you belong to?** | | | | | | |
| **Black or Black British**  □ Caribbean  □ African  □ Mixed White and Black Caribbean  □ Mixed White and Black African  □ Other Mixed Background | | | □ Any other black background (please state)  ………………………………  □ Other (please state)  ……………………………… | | | |
| **C4: We would like to know how long your family have lived in the UK. Please select which statement most applies to you:** | | | | | | |
| □ I was born in the UK and my parents were born in the UK  □ I was born in the UK but both of my parents were born outside the UK | | | □ I was born in the UK but one of my parents was born outside the UK  □ Neither I nor my parents were born in the UK | | | |
| If you were *born in the UK* PLEASE GO TO QUESTION C7 | | | | | | |
| **C5: What is your country of birth?** | | | | | | |
| ……………………………………………………….. | | | | | | |
| **C6: In which year did you move to the UK?** | | | | | | |
| …………………… | | | | | | |
| **C7: Which borough of London do you currently live in?** | | | | | | |
| ……………………………………………………….. | | | | | | |
| **C8: What is the main language you speak at home?** | | | | | | |
| □ English | | | □ Other (please state) ……………………………… | | | |

| **C9: What is the highest level of qualifications you have obtained?** | | | | | | |
| --- | --- | --- | --- | --- | --- | --- |
| □ No educational qualifications  □ GCSE/O-level/CSE  □ Vocational qualifications (e.g. NVQ)  □ A-levels or highers | | | □ University degree  □ Post graduate degree  □ Other qualification (please state)  ……………………………………………… | | | |
| **C10: What is your work status?** | | | | | | |
| □ Employed full-time  □ Employed part-time  □ Unemployed  □ Self-employed  □ Full-time homemaker/Housewife | | | □ Student  □ Retired  □ Disabled or too ill to work  □ Other (please specify)  ……………………………………………… | | | |
| **C11: Do you have any children?** | | | | | | |
| No | Yes, I have 1 | Yes, I have 2 | | Yes, I have 3 | | Yes, 4 or more |
| □ | □ | □ | | □ | | □ |
| **C12: What is your religion?** | | | | | | |
| □ Christian □ Muslim □ No religion | | | □ Other (please state) ……………………………………. | | | |
| **C13: How often do you attend religious services?** | | | | | | |
| Rarely or never | Few times a year | 1-3 times a month | | Once a week | At least twice a week | |
| □ | □ | □ | | □ | □ | |
| **C14: In general, you would say your health is:** | | | | | | |
| Poor | Fair | Good | | Very Good | | Excellent |
| □ | □ | □ | | □ | | □ |
| **C15: With regard to cigarette smoking, which is true for you?** | | | | | | |
| I have never smoked | I used to smoke occasionally,  but I quit | I used to smoke regularly,  but I quit | | I currently smoke occasionally | | I currently smoke regularly |
| □ | □ | □ | | □ | | □ |

***Thank you for taking part in this survey.***
