# Supplementary material for: Knowledge of cervical cancer and attendance at cervical cancer screening: a survey of Black women in London
Source: BMC Public Health. 2014 Oct 22;14:1096. doi: 10.1186/1471-2458-14-1096 (PMC4216339; doi:10.1186/1471-2458-14-1096)
Supplement: Supplementary file 3 — Additional file 3: Difficulties with recruitment. (DOCX 15 KB) [file 12889_2014_7195_MOESM3_ESM.docx]

**Supplementary material 3: Difficulties with Recruitment**

Reasons for deviating from original methodology

The original database was compiled from internet searches and magazine listings, and was prepared over a time period that predated the beginning of the recruitment process by up to a year. When the recruitment phase began, a number of issues arose which had not been foreseen. Firstly, it became evident that the contact details provided were frequently unreliable. Many of the phone numbers had been disconnected, and some were not correct and were actually private numbers rather than businesses. On visiting the salons, further problems arose. In some areas, the salons did not meet the criteria of being specialist black hair salons, and some salons turned out to be for men only, or unisex but with a predominantly male clientele. Other salons had closed down or could not be found.

To combat these issues the methodology was developed further to include an alternative strategy. The original plan of recruiting via the database was still followed as a priority, but once the database had been exhausted for each borough salons were recruited in person. This approach involved recruiting salons near to other successfully recruited salons, as salons seemed to be grouped in particular areas in each borough. It was generally easier to engage the salon staff in person, as trying to communicate the key information about the study over the phone had been challenging. By visiting salons to speak to the manager, it was possible talk to them without disrupting their working schedule, which was often very busy on a Saturday. Indeed some salons declined to be involved originally as they were too busy to speak on the phone.

Reasons for declining to be involved in the study and poor response rate

Some salons did not manage to recruit women as well as others. Salon staff suggested several reasons for poor response: 1) the data collection period was January-May and some salons felt that business was particularly quiet during this time, 2) some staff felt they were too busy to explain the study to clients, 3) some felt that cancer was not something their customers wanted to think or talk about and did not want to raise the topic, 4) In one area, a member of staff suggested that illiteracy might have been a problem, but that the women did not want to highlight this and so refused to take part in the study instead.
